# Practical Dentistry

**Published:** 1860-04

**Authors:** R. Arthur


### ARTICLE II.

### *Practical Dentistry.* By R. ARTHUR.

DURING the last twenty years there has been a great deal said about the advancement of the "Science" of dentistry. It has been truly urged that, as the teeth constitute a part of the animal organism they cannot become the subjects of disease independently of the rest of the system. That, with the rest of the body and the teeth, there are reciprocal morbid influences continually at work. No one, it has been urged, can be well qualified to practice dental surgery, unless he has a general acquaintance with anatomy, physiology, pathology and the nature and application of remedies. No one, at the present day, will be disposed to question the truth of this position. And yet, it may well be questioned whether the "Scientific" aspect of the necessary acquirements of a practicing dentist has not been held up too prominently before those who are about engaging in the practice of dentistry; whether the "Art" has not suffered in consequence.

While no intelligent dentist will undertake, for a moment, to deny the advantages of a large acquaintance with the human body, both in health and disease, as bearing upon his specialty, it must be conceded that dentistry is a manual art. The highest eminence in the practice of this art can never be reached independently of great manipulative dexterity. The disease known as dental caries, gives the dentist nearly all his work. He is by far the greater part of the time engaged in endeavoring to arrest its progress. Dental caries is produced by agents held in contact with a certain part of a tooth, and which effect a "solution of the continuity" of the part. The sole object of the efforts of the dentist is to restore this continuity, either by removing the broken and irregular surface, or by filling up the cavity formed with some material not subject to decomposition, and leaving the part in such condition that foreign substances will not be readily retained in contact with it. This constitutes the whole of the treatment of dental caries in the earlier stages. The material employed as a substitute for the lost dentine and enamel, best suited for the purpose, is now universally conceded to be gold. It must be so placed in the cavity of decay as to fill it up completely, so as to restore the part as nearly as possible, with some important modifications, to its original form. The preparation of the cavity, the shape given to the affected part of the tooth, the filling up with the gold, and the finishing of the filling, constitute together, an operation which must be performed in the most complete and perfect manner, to accomplish the object in view. In the treatment of dental caries, no assistance is derived from nature, as is the case with most operations in general surgery, in which the vital powers soon obliterate traces of the most bungling work on the part of the surgeon. The success of a "filling" depends entirely upon the perfection with which the work is done, subject, of course, to failure in some cases, from subsequent want of cleanliness on the part of the patient.

The operation to which I have been referring as employing nearly the entire time of a dentist, is in many cases of a most simple character. To prepare properly and fill many cavities of decay, require no more manual dexterity than it does to drive a nail badly into a board. In estimating the character, however, of many of these operations, it is necessary to consider the difficulty of access to many parts of the teeth in the mouth, the necessity of avoiding the pulp in the preparation of the cavity, the interference of the gum, the blood from parts it is impossible to avoid wounding, the mucous secretion, the saliva, the difficulty of seeing every part of the cavity during the operation, the frequent inability of the patient to bear steadily the unavoidable fatigue and pain, the frailty of the walls of the cavity, besides many other difficulties which it would require pages to describe, but which will occur to every practicing dentist, and when all these things are taken into the account, it is obvious that this operation requires the very highest order of manipulative skill. This kind of manual skill it is not in the power of every one to acquire; few indeed, the observation of every dentist of experience proves, possess it in a high degree, and when the natural ability does exist it has required long training and steady persevering labor to develope it as we have seen it developed in some very few operators at the present day, who stand prominently out among a number of practitioners, whose claims in this direction cannot be considered insignificant. If it be true that those persons who are endowed by nature with that rare talent of executing readily with their hands the conceptions of the mind, find themselves after years of faithful practice, steadily improving in their operations, and from year to year rendering better service to their patients, how much more necessary is it for the mass of men who turn their attention to this manual art to be made clearly to understand that their best and most earnest efforts are to be directed in this way, instead of leading them to believe that the theory of dental surgery is one of the most profound and intricate sciences known at the present day. I have no hesitation in asserting, and defy successful contradiction, that the theoretical knowledge necessary to qualify a practicing dentist for the duties he is called upon to perform, is comparatively easily acquired. While on the other hand the skill necessary to operate serviceably in cases which will present themselves almost every day in practice, can be acquired only by years of hard, careful, thoughtful, laborious effort.

Considerations of this character are forcing themselves upon many of those who are faithfully endeavoring to discharge the duties of their profession, in consequence of the almost daily, melancholy evidences of the entire worthlessness of a vast number of operations performed by dentists, young and old, occupying various positions, from the pushing advertising charlatan, to some of those enjoying the confidence of the best classes of the community. I refer to this matter not with any particular person or persons in view, not having the slightest disposition to make any of those covert but transparent petty personal flings, which have, to the great discredit of some of our periodicals, become so frequent of late. My object is rather to lead practicing dentists to the inquiry whether the advancement of dental surgery, in its most essential feature, has not been retarded by the stress which has been so much laid upon the necessity of the cultivation of the less practical branches of dental surgery.

There is, unquestionably, a great want in every part of the civilized world, so far as we have any acquaintance with their wants in this respect, of competent, careful and conscientious dental operators. For such men there is a large field every where. The harvest is truly abundant and the laborers few.

I am not at all disposed to deny that the number of good operators is increasing every year, but I am not sure, judging from my own observation, that the proportion greatly increases, and I have little doubt that the advance of the practical features of our useful vocation has been greatly retarded by an exaggeration of the scientific requirements necessary to make a competent dentist. A sickly struggle has been made for years, to place dentistry, as a "learned" profession, on a level with that of medicine. I mean that it has been urged that the actual intellectual preparatory requirements were equal. Indeed one would be disposed to conclude from the manner in which some of the "new lights" of dentistry express themselves, that it is not only a branch, but the most important branch of the healing art, in comparison with which, the minor branch of general surgery sinks into insignificance. It would be inferred from some of these learned gentlemen, that medicine is a branch of dentistry, but that, unfortunately for the latter, it has got the start in growth, and has most shamefully overshadowed it. That the exponents of the "science" of dentistry are most shamefully used by their fellow-doctors, whose ignorance prevents them from taking in an adequate idea of the scope of the profession. And many have been the frowns of indignation because of the unwarrantable refusal of this arrogant elder brother to take the younger scion into his household, and recognize his legitimacy; and because he cannot, on all occasions, succeed in concealing entirely his aversion to the assumption of a title which, in the healing art, he considered as already appropriated by himself.

No one can place a higher estimate upon the claims of dentistry to respect than I do; and no one, I am sure, would be more ready to exact it on all necessary occasions. No one can appreciate more highly than I do its just scientific claims and requirements; but I regret that exaggerated ideas of these claims and requirements have sometimes exposed us to just sarcasm, and have impaired our usefulness. It is always best that a man or class of men should correctly appreciate the position they occupy, and meet its requirements.

It cannot be denied that the great want of the community, even in this country, at the present day, in reference to dental surgery, is competent and faithful operators. I would urge, and if I could, would induce others who may have any influence, to urge upon students and those who have just commenced the practice of dentistry, the importance of directing their very best efforts to acquiring skill as operators. The true pathway to success and distinction lies in this direction. Let every young man labor with steady, patient perseverance to overcome the manual difficulties he must encounter in practice, and when he has succeeded in becoming a good operator, he will have accomplished more in his sphere as a dentist than if he had become a perfect master of the sciences which form the basis of the whole healing art.

If he allow his early days to pass with his attention diverted from this paramount demand upon his time, he will lose the golden moments when the hands and fingers more readily respond to the requirements of the will, than after they have become fixed in bad or imperfect habits.

There is no fear whatever, that by following this advice, a man of the right stamp will neglect other necessary qualifications. He will find from the very outset that the necessities of his practice will compel him to see; that while he *must* operate well, he can never be a thorough dentist if he is a *mere* operator.

And while the dentist keeps steadily before him the object and purpose of the business in which he is engaged, and keeps himself always fully up to its requirements, no one will rejoice more heartily or encourage him more earnestly than I will, to push his investigations and studies in a direction however far away it may seem to lead him, if they tend to throw light upon our specialty.

